# Intralesional acyclovir versus intralesional Hepatitis-B vaccine in treatment of resistant plantar warts: a randomized controlled trial

**DOI:** 10.1007/s00403-024-03001-4

**Published:** 2024-06-01

**Authors:** Khalid Gharib, Aya Taha, Mona Elradi

**Affiliations:** Dermatology, Venereology & Andrology Department, Zagazig University Hospitals, Zagazig, Egypt

**Keywords:** Acyclovir, HBV vaccine, Plantar, Warts

## Abstract

Treating plantar warts is still a challenging problem with a long list of diverse treatment options that none of them seems to be definitive. To evaluate the effectiveness of intralesional acyclovir versus intralesional Hepatitis-B vaccine (HBV) in treatment of multiple resistant plantar warts. Forty-eight patients with resistant plantar warts completed the study with no dropouts. They were randomized into 3 groups; group(A) receiving intralesional HBV, group (B) receiving intralesional acyclovir and group (C) receiving intralesional saline as a control group over 5 biweekly sessions or until wart clearance. Clinical outcome was assessed through sequential digital lesion photographing upon each visit. Treatment related adverse reactions were recorded. 43.8%, 37.5% & 18.7% of Groups A, B &C respectively showed a complete response. pain was obvious in 100% and 56.3% of cases receiving intralesional acyclovir and HBV respectively. Up to the 6 month follow up period, none of the complete responders in all groups returned with a recurrence. Both acyclovir and HBV showed comparable efficacy and seem to be promising options for treating plantar warts being safe, affordable, and theoretically safe in immunocompromised cases.

## Introduction

Plantar warts exhibit a high propensity for being resistant to treatment and tendent for recurrence. Being painful, in most instances, they impair ambulation and hence affect patients’ quality of life [1]. Moreover, plantar warts show a lower response rate than warts at any other anatomical site [2]. There is a lack of evidence for nearly all available treatments with no one standardized option with an expected and a consistent clinical efficacy for all cases [1].

Topical keratolytics and cryotherapy were and still are the two most widely used options for treating plantar warts. Both are associated with a multitude of adverse effects and a highly probable recurrence [1]. Recently, there’s an emerging trend of various intralesional injections either immunologic e.g. Candida antigen [3, 4] or non-immunologic e.g. vitamin D3 [5] that are promising but not sufficiently represented in large studies [6].

Intralesional antigen therapy has, by far, become one of the safest and most effective therapeutic modalities that can effectively clear not only primary but distant warts as well with a low incidence of recurrence. It elicits a delayed-type hypersensitivity response through mounting of T-helper1 (Th1) cytokines e.g. IL-2, IFN-γ & TNF-α that exert an antiviral effect against Human papilloma virus; causative agent of warts [7].

Hepatitis-B vaccine (HBV) is a non-live vaccine that has been recently introduced into the routine vaccination schedule of infants and adolescents till the age of 18. Being non-live, it’s also theoretically safe for elderly and immunocompromised. Three generations have been introduced into the market, the second of which is developed by recombinant DNA technology and is most widely used [8, 9].

Hepatitis-B vaccine is known to promote both a humoral and a cellular immune response. It has recently been investigated as a new immunotherapeutic option for common warts with a low rate of complete response being injected intra-lesionally [8.9] and a relatively higher response rate when injected intra-muscularly [8].

The viral origin of warts suggests than an antiviral drug with a proven efficacy against DNA viruses, such as acyclovir, may be a potential option for treatment [6]. Back in 1982, Bauer reported a single patient with cleared recalcitrant plantar warts after local application of acyclovir cream [9]. A year later, Pechman demonstrated the successful use of acyclovir ointment in a patient with refractory plantar warts [10].

Cases with resistant plantar warts disappearing after a course of oral acyclovir for a concomitant herpes zoster were also reported [11, 12]. In 2022, Elsayed et al. evaluated the efficacy of intralesional acyclovir injection for treating common warts and achieved a complete response in 52.6% of their patients [13]. Herein and based on the abovementioned findings, investigating the efficacy of both HBV and acyclovir in treating plantar warts sounds legitimate.

## Patients and methods

Forty-eight patients with resistant or recurrent plantar warts were included. A written informed consent was obtained from all patients after the study was approved by the institutional review board of Zagazig University.

All patients reported failure of at least one previous therapeutic course and had a washout period of at least 3 months from the last treatment they had for their warts. Any patients with acute illness, fever, pregnant and lactating females were excluded from the study. All patients enrolled for intralesional acyclovir injection underwent kidney function tests to exclude any renal disorder. All patients enrolled for intralesional HBV had no prior vaccination with HBV to omit the notion of enhanced clinical response in previously vaccinated patients [8].

Warts were thoroughly clinically assessed according to the CWARTS tool [14] to define their different morphological characteristics (arrangement, level, aspect, border sharpness, presence of black dots, size and color) and assess their relation to response therapy. All included patients had not any distant warts.

### Methods

Patients were randomly distributed into 3 groups; group (A) received intralesional HBV, Group (B) received intralesional acyclovir and group (C) received intralesional saline as a control group. Patients in group A were injected with a total of 0.3 ml of HBV (GeneVac-B, Serum Institute of India, Ltd., India) at a dose of 0.1 ml injected using an insulin syringe into the largest 3 warts.

Patients in group B were injected with a total of 0.3 ml of acyclovir (Zovirax vials, Galaxosmithkline (GSK), UK), at a concentration of 70 mg/ml, at a dose of 0.1 ml injected in the same manner as above. Patients in group C received a total of 0.3 ml isotonic saline injected intra-lesionally in the same previous manner. All patients underwent biweekly sessions until complete cure or a maximum of 5 sessions were reached.

Clinical response was assessed by two blinded dermatologists at each visit. Clinical digital photographs of the lesions were taken at baseline, each visit and at the follow up visit 6 months after their last visit. Response was regarded either as a complete response by total disappearance of the warts and return of the usual skin markings, a partial response by regression of wart size by about 50–90% and a no response is the regression was between 0 and 49%.

All patients signed a written informed consent after thoroughly explaining the procedure done, side effects expected and allowed the use of their photos for publication.

### Statistical analysis

All data were collected, tabulated, and statistically analyzed using SPSS Statistics for Windows (Version 23.0. Armonk, NY: IBM Corp.). Mann Whitnney-U test: was used to compare between two groups of non-normally distributed variables. Kruskall Wallius test: was used to compare between more than two groups of non-normally distributed variables. Percent of categorical variables were compared using Chi-square test or Fisher Exact test. All tests were two sided. p-value < 0.05 was considered statistically significant.

## Results

This study included an overall of 48 patients: 17 females and 31 males with no statistical difference in age or gender among studied groups. There was no statistically significant difference between groups regarding various clinical data such as wart duration, previous therapies, whether warts were recurrent or not and the different morphological characteristics assessed using the CWARTs tool.

Regarding the therapeutic response, there was no statistically significant difference between groups A and B, but both showed a statistically more powerful response than group C injected with saline. 7 (43.8%), 6 (37.5%) and 3(18.7%) out of 16 patients in groups A, B and C respectively achieved a complete response (Table 1).

**Table 1:** Therapeutic response among studied groups

Clinical response	Group A *N* 16	Group B *N* 16	Group C N16	P1	P2	P3
	*N* (%)	*N* (%)	*N* (%)			
Complete response	7(43.8)	6(37.5)	3(18.7)			
Partial response	3(18.7)	6(37.5)	0(0)	0.47	0.003*	0.025*
No response	6(37.5)	4(25.0)	13(81.3)			

**χ**^**2**^ :Chi square test

P value > 0.05 statistically non-significant, *P value < 0.05 statistically significant

P1 (comparing groups A & B), P2 (comparing groups B& C), P3 (comparing groups A & C)

**Fig. 1:**
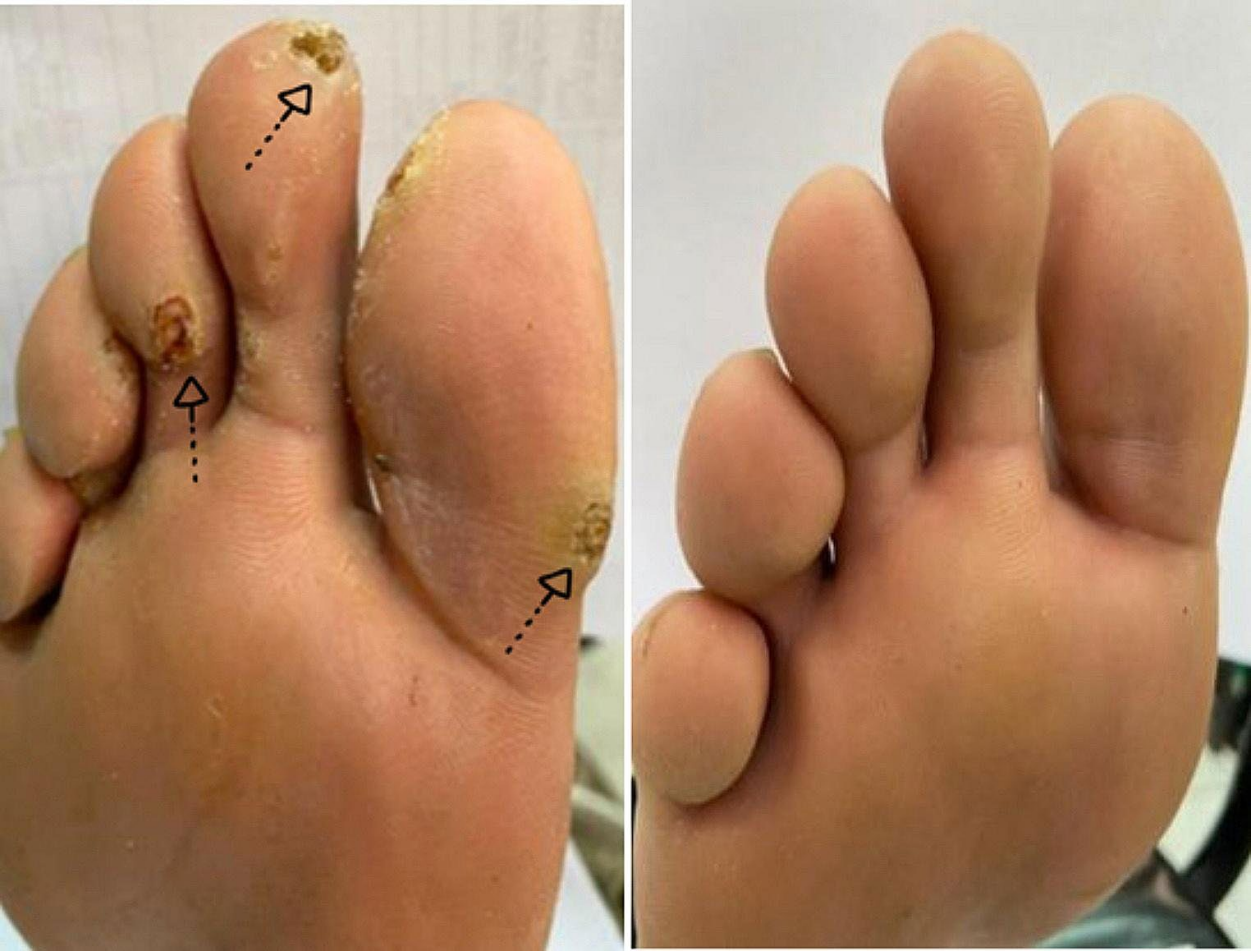
Left foot of a 30-year old male patient with multiple resistant planter warts completely cured after intralesional HBV injection

**Fig. 2:**
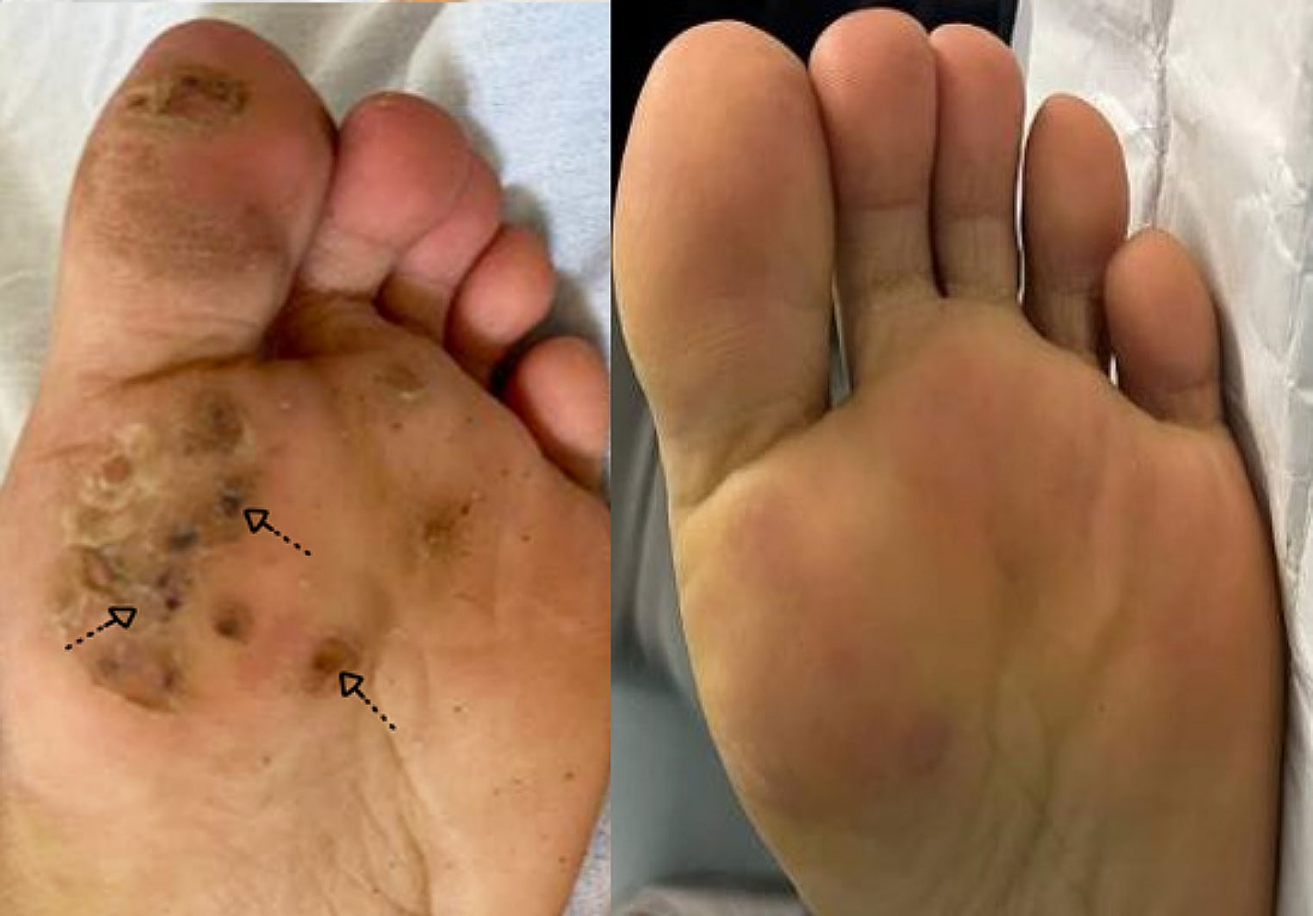
Right foot of a 22-year old male patient with multiple resistant planter warts completely cured after intralesional acyclovir injection

**Fig. 3:**
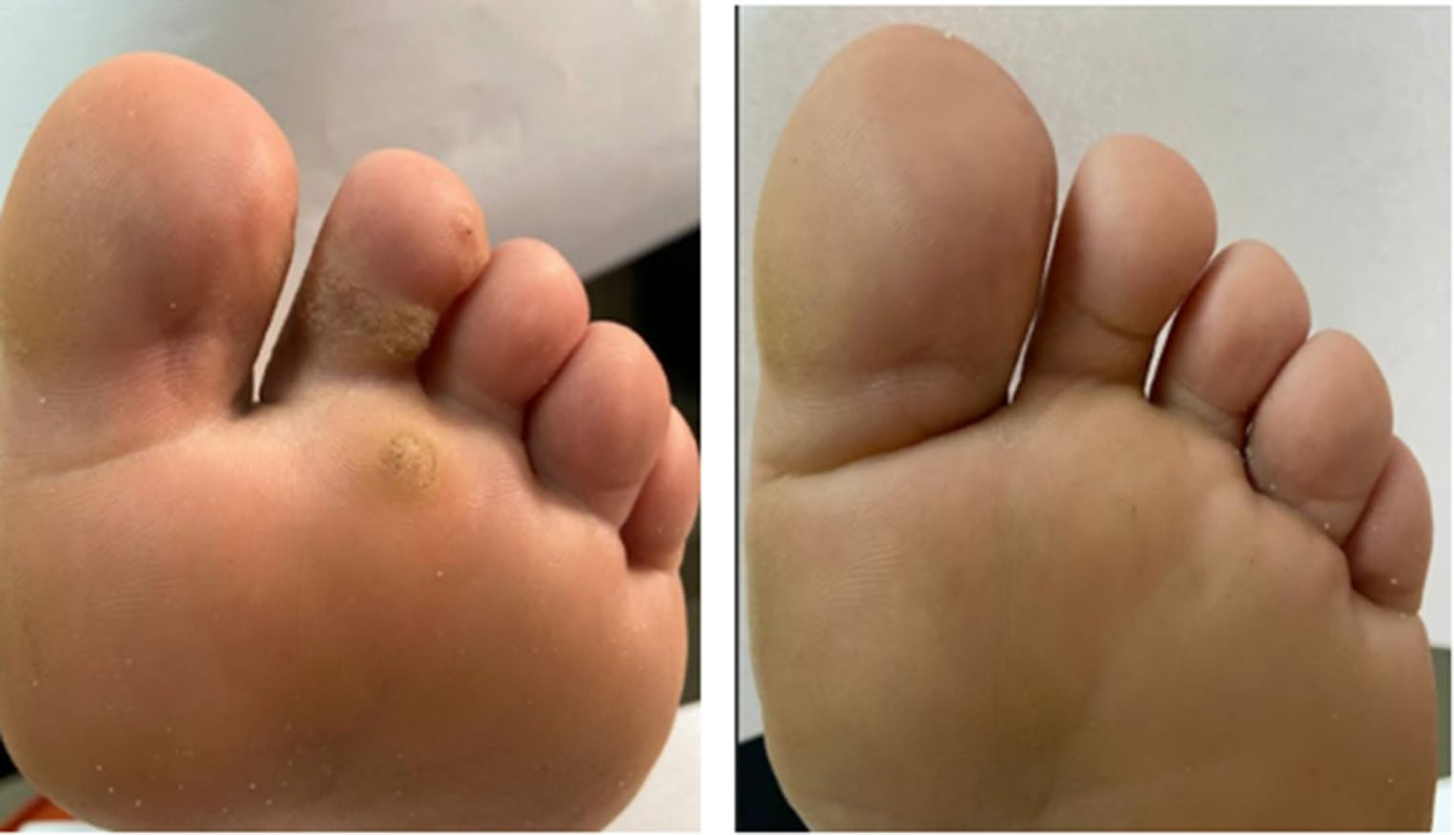
Left foot of a 20-year old female patient with multiple resistant planter warts completely cured after intralesional saline injection

Out of the 7 patients exhibiting a complete response in response to HBV (Figs. 1), 1 patient reached this complete cure after only 1 session and 2 other patients after 3 sessions. Out of the 6 patients exhibiting the complete response to acyclovir (Figs. 2), 1 patient reached this complete cure after 2sessions and 1 patient after 3 sessions. 3 other patients reached it after 4 sessions. The 3 patients exhibiting complete cure after saline injection received only 3 sessions (Fig. 3).

Pain during injection was reported by all patients receiving acyclovir and most of them reported it to be severe. 56.3% of patients receiving HBV complained of pain during injection. Intralesional injection of saline was reported painful by 37.5% of patients only with a statistically significant pain incidence in groups A&B than the control group. A mild allergic reaction was reported in only 3 patients receiving HBV.

Results showed no statistically significant relation between response to HBV and any of the demographic, clinical and warts’ morphological variables except for warts with a smooth aspect and those with that are solitary not confluent were statistically more responsive. The same goes for response to acyclovir except for warts previously surgically removed were less responsive to acyclovir. As for saline, the 3 cases that showed the response to intralesional saline had their warts previously treated with some kind of immunotherapy.

Upon follow up, none of the patients who showed complete response to any of the used lines returned with recurrence after a 6 month follow up period.

## Discussion

Resistance to therapy and recurrence are the two most widely encountered problems when dealing with plantar warts. Finding a therapy that is effective, available with a low side effect profile is still researchable. Intralesional immunotherapy for wart treatment still has an uncertain mechanism of action but is repeatedly mentioned to be associated with an induced cell mediated immune response leading to upregulation of Th1 cytokine profile e.g. IFN-, TNF-, ILs 2& 12 mediating a cytotoxic response against virally infected cells not only in the injected warts but in non-injected distant warts as well [15].

Hepatitis-B vaccine (HBV) is a very recently introduced immunotherapeutic line for treating warts being safe, readily available, exceedingly stable and linked to promoting a strong immune response [8, 16]. The proven efficacy of acyclovir against DNA viruses strongly recommends it as a potential antiviral line in treating warts [6].

Up to the best of our knowledge, this randomized controlled trial is the first study to evaluate the effectiveness of intralesional HBV, as an immunotherapeutic agent, versus intralesional acyclovir, as an antiviral agent, in treating resistant plantar warts. Both lines were statistically more effective than intralesional saline with no statistically significant difference in the clinical response between both groups.

Using second generation HBV, Nofal et al. in 2021 achieved a complete response in only 20.7% of patients with common warts treated with intralesional HBV [16]. In 2022, he reported compete wart clearance in 23.3% and 50% of patients with common warts using intralesional and intramuscular HBV respectively [8].

Compared to the response rates of this study, 43.8% of patients were completely cured indicating superior results for intralesional HBV in treating plantar warts. Nofal et al. also observed that the response was higher and earlier in previously vaccinated patients with HBV [8]. According to this finding, non-vaccinated patients only were included to omit this factor. Children and adults below 30 showing a more robust immune response to the vaccine than older people according to [8] might explain the relatively higher response rate, in this study, since the mean age of complete responders was 25.1 ± 2.7.

On another hand, in comparison to other immunotherapeutic agents, the success rates encountered in this study were lower than those of using Candida antigen. Khozeimeh et al. in 2017 and Nofal et al. in 2022 achieved complete response in 76.7% and 84% of patients complaining of recalcitrant plantar warts respectively [3, 17]. Using MMR vaccine in treating recalcitrant plantar warts, Rezai et al. achieved complete response in 65.2% of patients [18].

The lower response rate using HBV may be due to the different nature and reactivity of the injected agents i.e. live vaccine such as MMR vaccine are more immunogenic than non-live vaccines such as HBV. However, an advantage goes for HBV, being non-live, that it can be safely used in pregnant and immunocompromised individuals. Moreover, the risk of allergic reactions, commonly encountered with candida antigen, is minimal if not null [19].

In 1982, Bauer reported clearance of recalcitrant plantar warts after local application of acyclovir cream in a single case report [9]. A year later, Pechman demonstrated the successful use of acyclovir ointment in a patient with refractory plantar warts [10]. In 2005, Tandeter and Tandeter reported complete resolution of resistant plantar warts after oral intake of valacyclovir 1 gm for coexistent herpes zoster [11]. Similarly, Bagwell et al., described a case of recalcitrant plantar warts that disappeared 10 days after acyclovir therapy for herpes zoster [12].

In 2022, Elsayed et al. reported complete clearance of common warts in 52.6% of their patients using intralesional acyclovir while 37.5% of patients with plantar warts, in this study, were complete responders. These different response rates may be basically related to the different type of wart treated in the two studies. Differences in warts characteristics and number of patients treated may be also partially responsible [13].

Worthy to mention that 2 out of the 3 patients who responded completely to intralesional saline injection received an intralesional immunotherapy months before the start of the study. Thus, this response might be explained as a delayed immune response or may be suggestion played a role [20].

Regarding the side effects of the used modalities, sever pain observed in all patients who received intralesional acyclovir can pose a limitation for it use. Yet, its results are still promising. Therefore, investigating another route of administration is recommended. Further studies on larger populations in comparison with other traditional methods and different wart types are recommended as well. In conclusion, we believe these results are solid and very well scientifically based to consider those two available and safe modalities as potential effective options for treating plantar warts.

## Data Availability

No datasets were generated or analysed during the current study.
